# Alveolar Fluid Clearance in Pathologically Relevant Conditions: *In Vitro* and *In Vivo* Models of Acute Respiratory Distress Syndrome

**DOI:** 10.3389/fimmu.2017.00371

**Published:** 2017-04-07

**Authors:** Laura A. Huppert, Michael A. Matthay

**Affiliations:** ^1^Department of Medicine, University of California, San Francisco, CA, USA; ^2^Departments of Medicine and Anesthesia, UCSF School of Medicine, Cardiovascular Research Institute, San Francisco, CA, USA

**Keywords:** acute respiratory distress syndrome, alveolar fluid clearance, mesenchymal stem (stromal) cells, pulmonary edema, vectorial ion transport

## Abstract

Critically ill patients with respiratory failure from acute respiratory distress syndrome (ARDS) have reduced ability to clear alveolar edema fluid. This reduction in alveolar fluid clearance (AFC) contributes to the morbidity and mortality in ARDS. Thus, it is important to understand why AFC is reduced in ARDS in order to design targeted therapies. In this review, we highlight experiments that have advanced our understanding of ARDS pathogenesis, with particular reference to the alveolar epithelium. First, we review how vectorial ion transport drives the clearance of alveolar edema fluid in the uninjured lung. Next, we describe how alveolar edema fluid is less effectively cleared in lungs affected by ARDS and describe selected *in vitro* and *in vivo* experiments that have elucidated some of the molecular mechanisms responsible for the reduced AFC. Finally, we describe one potential therapy that targets this pathway: bone marrow-derived mesenchymal stem (stromal) cells (MSCs). Based on preclinical studies, MSCs enhance AFC and promote the resolution of pulmonary edema and thus may offer a promising cell-based therapy for ARDS.

## Introduction

Pulmonary edema is the abnormal accumulation of fluid in the interstitium and air spaces of the lungs, which leads to impaired gas exchange and respiratory failure. Pulmonary edema can develop from increased pulmonary vascular pressure from left heart failure (cardiogenic pulmonary edema) (1) or from lung parenchymal damage from increased endothelial and epithelial permeability (non-cardiogenic pulmonary edema) (2). In both cases, the mechanism for the resolution of alveolar edema is the same: active ion transport across the alveolar epithelium creates an osmotic gradient that drives alveolar fluid clearance (AFC) (3). In the presence of acute lung endothelial and epithelial injury, there is complexity in describing the forces responsible for lung fluid clearance, meaning removal of edema from the lung itself. Net AFC does depend on an intact epithelial barrier that can transport ions from the apical to the basolateral surface and create a mini-osmotic gradient for alveolar fluid absorption. If transvascular fluid flux is increased across lung endothelium from increased pressure or increased permeability, then the rate of AFC will be reduced. Also, net lung fluid clearance will be less. Lung lymphatics do remove edema fluid in either hydrostatic or increased permeability lung edema, but they cannot entirely compensate for an increase in transvascular fluid flux or impaired AFC.

Acute respiratory distress syndrome (ARDS) is a syndrome of acute respiratory failure caused by non-cardiogenic pulmonary edema. The most common cause of ARDS is bacterial or viral pneumonia (4). Sepsis due to non-pulmonary sources, trauma, aspiration, pancreatitis, transfusion reactions, and drug reactions can also lead to ARDS (4). Criteria for the diagnosis of ARDS have changed over time, but the current definition includes arterial hypoxemia with PaO_2_/FiO_2_ ratio less than 300 mmHg, bilateral radiographic opacities, without evidence of that is not fully explained by cardiac failure or fluid overload (5). The mortality of ARDS is approximately 25–40% (6), and treatment remains primarily supportive with lung protective ventilation and a fluid conservative strategy (7).

Because ARDS has a broad clinical phenotype, it has been challenging to translate cell and animal studies to pharmacologic therapies that reduce human morbidity and mortality. Nonetheless, *in vitro* and *in vivo* studies have produced important insights about the pathogenesis of this condition, paving the way for targeted therapeutics. This review will focus on: (1) mechanisms that mediate the clearance of pulmonary edema in the uninjured lung, (2) why AFC is reduced in ARDS, resulting in the accumulation of pulmonary edema fluid, and (3) one potential treatment for ARDS with a cell-based therapy that may accelerate the rate of AFC.

## Pulmonary Edema Fluid Clearance in the Uninjured Lung

**Figure 1:**
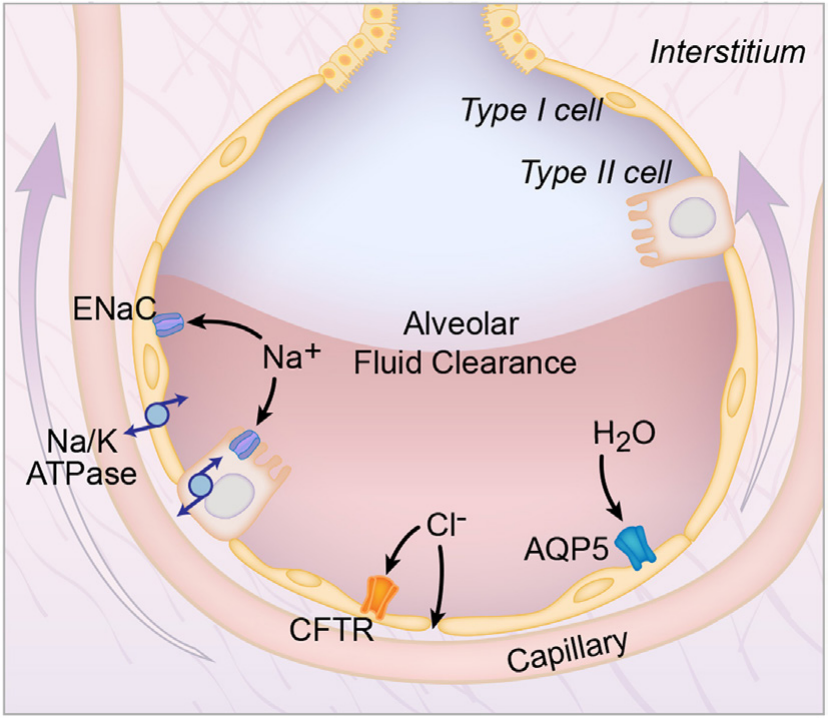
**Alveolar fluid clearance pathways**. Shown are the interstitial, capillary, and alveolar compartments, with pulmonary edema fluid in the alveolus. Both type I (yellow) and type II (orange) alveolar cells are involved in transepithelial ion transport. Sodium (Na^+^) is transported across the apical side of the type I and type II cells through the epithelial sodium channel (ENaC), and then across the basolateral side *via* the sodium/potassium ATPase pump (Na/K-ATPase). Chloride (Cl^−^) is transported *via* the cystic fibrosis transmembrane conductance regulator (CFTR) channel or by a paracellular route. Additional cation channels also transport ions across the alveolar epithelium (not shown). This vectorial ion transport creates an osmotic gradient that drives the clearance of fluid. Specifically, water (H_2_O) moves down the osmotic gradient through aquaporin channels, such as aquaporin 5 (AQP5) or *via* an intracellular route (not shown).

Before discussing AFC in ARDS, it is first important to review how pulmonary edema fluid is cleared in the uninjured lung. In the uninjured lung, vectorial ion transport across the alveolar epithelial cells creates an osmotic gradient that drives fluid from the airspaces into the lung interstitum (Figure 1). It was initially thought that alveolar epithelial type II cells were the primary cell responsible for vectorial ion transport, but subsequent studies demonstrated an important role for type I cells as well (8). The transport of sodium ions is the most important driver for the generation of the osmotic gradient: sodium is transported through the sodium channel (ENaC) on the apical surface and then by the Na/K ATPase on the basolateral surface into the lung microcirculation (9, 10). Knockout of the alpha-subunit of ENaC in mice resulted in the inability to remove lung fluid at birth with subsequent respiratory failure and death (9). In addition, non-selective cation channels, cyclic nucleotide-gated channels, and the cystic fibrosis transmembrane conductance regulator chloride channel also contribute to the creation of the osmotic gradient (3, 11). Aquaporins facilitate the movement of water across the epithelial surface, but are not required for fluid transport (12).

This system of active ion-driven alveolar fluid reabsorption is the primary mechanism that removes alveolar edema fluid under both physiologic and pathological conditions (9, 13, 14). However, in the setting of ARDS, the capacity to remove alveolar edema fluid is reduced, which is termed impaired AFC. A reduction in the rate of AFC in ARDS correlates with decreased survival (15, 16). Therefore, it is critical to better understand why AFC is reduced in ARDS to better understand the pathogenesis of this condition.

## Pulmonary Edema Fluid Clearance in ARDS

Multiple mechanisms explain why AFC is reduced in ARDS. First, both hypoxia and hypercapnia impair AFC. ENaC transcription and trafficking is downregulated and Na/K-ATPase functions less efficiently under states of low oxygen or high carbon dioxide, in part, because reactive oxygen species trigger endocytosis and cell necrosis (17–19). Therefore, supplemental oxygen and correction of hypercapnia can enhance the resolution of alveolar edema (17).

Second, biomechanical stress can reduce AFC. High tidal volumes and elevated airway pressures injure the alveolar epithelium, inducing cell death and inflammation, which reduces AFC (20). If pulmonary hydrostatic pressures are elevated, the rate of AFC is also reduced. These findings help explain the success of lung protective ventilation strategies and conservative fluid strategies in reducing the morbidity and mortality of ARDS (21, 22).

Third, ARDS pulmonary edema fluid contains high levels of pro-inflammatory cytokines including IL-1β, IL-8, TNFα, and TGFβ1 (23–25). Under controlled conditions, this inflammatory response is important for pathogen clearance. However, when excessive levels of cytokines are present, they instead can cause alveolar injury and decreased AFC (26–29). Moreover, once the epithelial barrier is breeched and alveolar fluid is released, components of the alveolar fluid may be recognized to induce downstream inflammatory and immune responses (30). There are no current therapies that directly target this pathway in the treatment of ARDS, although lung protective ventilation itself reduces pro-inflammatory cytokines such as IL-6 and IL-8 (4, 31). The remainder of this review will summarize our current understanding of alveolar ion transport in ARDS based on pathologically relevant *in vitro* and *in vivo* models, and discuss one potential new therapy that targets this pathway.

**Figure 2:**
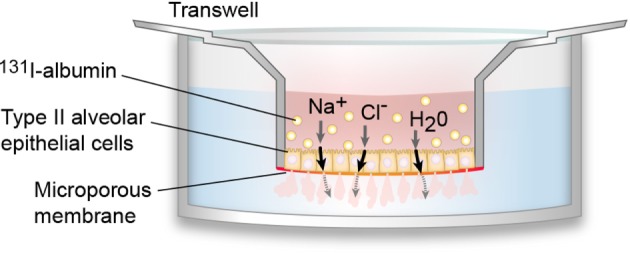
***In vitro* model of polarized human alveolar type II epithelial cells**. In 2006, Fang et al. developed an *in vitro* model of the polarized human alveolar epithelial surface, which has been used in multiple subsequent studies of alveolar fluid clearance (AFC) (32). To create this model, type II alveolar epithelial cells are isolated from human donor lungs and cultured on a collagen-I coated 24-well plate where they formed tight monolayers. Pulmonary edema fluid (pink), which contains water (H_2_O), sodium ions (Na^+^), chloride ions (Cl^−^), as well as other ions and proteins, is mixed with ^131^I-albumin (yellow circles) and introduced to the apical compartment. Pulmonary edema fluid is able to cross the alveolar cell monolayer, but ^131^I-albumin cannot cross, so it is possible to calculate AFC by measuring the change in ^131^I-albumin concentration between the apical and basal compartments.

In 2006, Fang et al. developed a model of a polarized human alveolar type II cell that facilitated *in vitro* studies of AFC (32) (Figure 2). Using this model, Lee et al. found that transepithelial fluid transport is less effective in the presence of ARDS edema fluid and found that there are increased levels of cytokines and decreased levels of ion transport proteins in the presence of ARDS edema fluid compared to a plasma control (33). These data support the hypothesis that cytokine expression is increased in alveolar epithelium during ARDS, resulting in a decreased expression of alveolar ion channels and accumulation of alveolar edema fluid. In addition, the inflammatory edema fluid can also cause alveolar cell injury and necrosis, resulting in altered epithelial tight junctions (34, 35). The loss of tight junctions can negate the osmotic gradient, offsetting the effects of vectorial ion transport.

Subsequent *in vivo* studies expanded upon these findings and demonstrated that net AFC was reduced under clinically relevant pathologic conditions in animal models. In sheep, live *Pseudomonas aeruginosa* decreased AFC at both 4 and 24 h, an effect, which was associated with decreased AFC (36). In a mouse model of influenza pneumonia, the authors demonstrated that there was decreased AFC due to inhibition of the ENaC epithelial sodium channel (37). These and other studies confirmed that AFC is reduced in lung injury through decreased efficiency of alveolar ion channels as well as altered permeability of the normally tight alveolar epithelium.

## Mesenchymal Stem (Stromal) Cells (MSCs) as a Promising Therapy for ARDS

The laboratory-based investigations described above significantly advanced our understanding of ARDS pathophysiology, paving the way for targeted molecular therapies to improve the clinical treatment of patients with ARDS. MSCs are one such promising new cell-based therapy, which we will discuss in this section.

Mesenchymal stem (stromal) cells are bone marrow-derived cells that can differentiate *in vitro* into chondrocytes, osteoblasts, and adipocytes, although they do not have true stem cell properties *in vivo* (38). MSCs secrete paracrine factors that can decrease inflammation and enhance endothelial and epithelial repair (39). Several groups are studying the therapeutic potential of MSCs in sepsis (40, 41), diabetes (42), myocardial infarction (43), hepatic failure (44), and acute renal failure (45, 46). It was hypothesized that MSCs might also be beneficial in the treatment of ARDS.

To test this hypothesis, several groups studied whether MSCs could reduce the severity of lung, kidney, and brain injury in preclinical models (47). In 2007, Gupta et al. reported that treatment with MSCs improved survival and reduced pulmonary edema in *Escherichia coli* endotoxin-induced lung injury in mice (48). Subsequent studies showed that MSCs attenuated lung injury in mice and in *ex vivo* human lungs injured with live bacteria (49, 50). MSCs also enhance bacterial clearance and improve survival in mouse and rat models of sepsis (41, 51), and they have beneficial effects in ventilator-induced acute lung injury (52). Based on this preclinical data, phase 1 and 2 clinical trials are currently testing MSCs as a therapy for ARDS (53).

Given the potential therapeutic benefit of MSCs in the treatment of ARDS, it is important to understand their mechanism of action and several possible mechanisms have been implicated to date. In 2010, Fang et al. used SiRNA knockdown of paracrine soluble factors in the setting of MSC treatment *in vitro* in cultured human type 2 cells and found that angiopoietin-1 secretion was partially responsible for the beneficial effect of MSCs (54). Subsequent studies suggested that interleukin-1 receptor antagonist and growth factors such as keratinocyte growth (KGF) factor may also be involved in this process (52). KGF can upregulate AFC in *ex vivo* human lungs injured by endotoxin (55). A 2015 study demonstrated that lipoxin A4, a pro-resolving lipid mediator, is upregulated in the presence of MSCs, suggesting that this molecule may be important for MSC-mediated resolution of lung injury (56). Other studies indicated that the therapeutic effects of MSCs may also mediate the release of microvesicles, which are involved in cell–cell communication (57) or due to mitochondrial transfer (58). Thus, several mechanisms may explain MSC-mediated resolution of lung injury, and further studies are needed to fully characterize this process.

## Discussion

The importance of alveolar ion transport in AFC has been established, but further work is needed to better characterize this process. For example, type I alveolar cells participate in apical–basolateral fluid transport (8, 59), but there is no suitable cell culture model for type I cells so the physiology of these cells is not as well understood. In addition, sodium transport has been well characterized, but the contribution of transport of other ions is not as clear. Future experiments are needed to clarify the role of type I versus type II alveolar cells and the roles of additional ion channels in alveolar fluid transport.

The *in vitro* and *in vivo* models of ARDS have enhanced our understanding of ARDS pathophysiology. Not only are these models useful for the study of ARDS but measurements of AFC and paracellular permeability can also be used to better understand other pulmonary conditions. For example, Chan et al. (60) compared the extent to which avian influenza A (H5N1) virus and seasonal influenza A (H1N1) virus impair AFC and protein permeability using the transwell model first used in the ARDS models (60). The authors found that avian influenza A (H5N1) virus causes a more severe reduction in alveolar protein transport than the seasonal influenza A (H1N1) virus, mimicking its greater clinical severity. Future work can use these models to better understand how AFC is affected in other pulmonary pathologies as well.

Preliminary preclinical experiments have suggested that MSCs promote the resolution of alveolar edema fluid. It is possible that MSCs act directly on alveolar ion channels *via* cell–cell interactions or indirectly *via* paracrine factors; future experiments are needed to clarify their mechanism of action in the lung. If MSCs indeed promote AFC, they may serve as a promising cell-based therapy for ARDS.

## Summary

In this review, we have discussed how vectorial ion channels in alveolar epithelium generate an osmotic gradient that drives AFC in both physiologic and pathologic conditions. Both AFC and paracellular permeability can be measured using *in vitro* and *in vivo* models of ARDS, and these studies indicate that vectorial ion transport is less effective in injured lungs than in uninjured lungs. Recent studies suggest that MSCs interact with alveolar epithelium and ion channels to increase AFC and thus may serve as a promising treatment for ARDS.

## Author Contributions

LH and MM composed this mini-review manuscript together. LH is the first author.

## Conflict of Interest Statement

The authors declare that the research was conducted in the absence of any commercial or financial relationships that could be construed as a potential conflict of interest.
